# A longitudinal study of the effect of short-term meditation training on functional network organization of the aging brain

**DOI:** 10.1038/s41598-017-00678-8

**Published:** 2017-04-04

**Authors:** Francesca A. Cotier, Ruibin Zhang, Tatia M. C. Lee

**Affiliations:** 1 Laboratory of Neuropsychology, The University of Hong Kong, Pok Fu Lam, Hong Kong; 2 Laboratory of Cognitive Affective Neuroscience, The University of Hong Kong, Pok Fu Lam, Hong Kong; 3 The State Key Laboratory of Brain and Cognitive Sciences, The University of Hong Kong, Pok Fu Lam, Hong Kong

## Abstract

The beneficial effects of meditation on preserving age-related changes in cognitive functioning are well established. Yet, the neural underpinnings of these positive effects have not been fully unveiled. This study employed a prospective longitudinal design, and graph-based analysis, to study how an eight-week meditation training vs. relaxation training shaped network configuration at global, intermediate, and local levels using graph theory in the elderly. At the intermediate level, meditation training lead to decreased intra-connectivity in the default mode network (DMN), salience network (SAN) and somatomotor network (SMN) modules post training. Also, there was decreased connectivity strength between the DMN and other modules. At a local level, meditation training lowered nodal strength in the left posterior cingulate gryus, bilateral paracentral lobule, and middle cingulate gyrus. According to previous literature, the direction of these changes is consistent with a movement towards a more self-detached viewpoint, as well as more efficient processing. Furthermore, our findings highlight the importance of considering brain network changes across organizational levels, as well as the pace at which these changes may occur. Overall, this study provides further support for short-term meditation as a potentially beneficial method of mental training for the elderly that warrants further investigation.

## Introduction

There has been considerable work evidencing both cognitive and neural decline in elderly populations^1, 2^. Studies have shown deterioration of performance in a number of cognitive tasks^3^, as well as significant changes in brain structure and function^4^. Numerous training programs have been developed to counter this age-related decline^5^. Whilst the effectiveness of some of these programs has yet to be verified^6^, several of them have been clearly shown to incur positive effects^7^. Thus, these suggest that the elderly brain may still undergo neuroplastic changes.

One form of training that has gained increasing popularity in recent years is meditation^8^. The effectiveness of meditation in reducing cognitive decline in elderly individuals has been examined extensively, and improvements in several areas of cognitive function have been found^9^. Researchers have also begun to investigate the impact of mediation on the elderly brain. There is evidence that elderly meditators do not suffer from the same extent of reductions in gray matter volume as their age-matched controls^10, 11^; reductions in age-related decline regarding fractional anisotropy in several white matter fibre tracts have also been found^12^. Shao, *et al*.^13^ explored the impact of an 8-week meditation training on the elderly, and found increased positive connectivity between the pons and PCC/precuneus. Together, these findings suggest that meditation incurs significant neurocognitive effects on the aging brain.

One approach to examining the impact of aging on the brain is through graph-based analysis. Graph theory postulates that the structure of the brain is dependent on a trade-off between minimizing cost and maximizing efficiency^14^. A number of studies have examined age-related changes in network-organization and, in general, point towards reductions in small worldness, or a less ‘optimal’ trade-off between cost and efficiency with increasing age^15, 16^.

Few studies have examined changes in brain functional organization in relation to meditation in elderly populations. Most recently, Jao, *et al*.^17^ compared network topology of Taoist meditators during states of resting and meditation respectively. Interestingly, no differences were found at a global level. However, at the level of individual nodes and hubs, significant expertise-dependent reorganisation was seen during meditation particularly in areas of the default mode network (DMN). Whilst this study points towards meditation state-related changes in brain network organization, conclusions that can be made are limited by the absence of a control group from the study. On the other hand, Gard, *et al*.^9^ employed an age-matched control group to examine age-related changes in terms of fluid intelligence and brain functional organization (e.g. small worldness) in expert yoga and meditation practitioners. The combined results indicated that meditators and yoga practitioners have a relatively small-world like organizational structure compared to the controls. Again, whilst this study provides insight into how meditation may impact elderly brain configuration, given the cross-sectional design, it is possible that the observed differences already existed prior to the meditation. In order to address these issues, a prospective longitudinal design was employed to explore how an eight-week meditation training program shapes network configuration in the elderly brain, as compared with training in relaxation. Specifically, we examined brain network configuration changes in response to meditation at three levels; 1) a global level (whole brain topology), 2) an intermediate level (intra-module and inter-module connectivity) and 3) at a local level (nodal strength). Based on the research thus far, we hypothesized that at a global level the meditation group will show significantly more small world-like organizational structure compared to controls. At intermediate and local levels, significant network reorganization (particularly within the DMN), specific to meditation, was also predicted.

## Results

### Study Population

**Figure 1:**
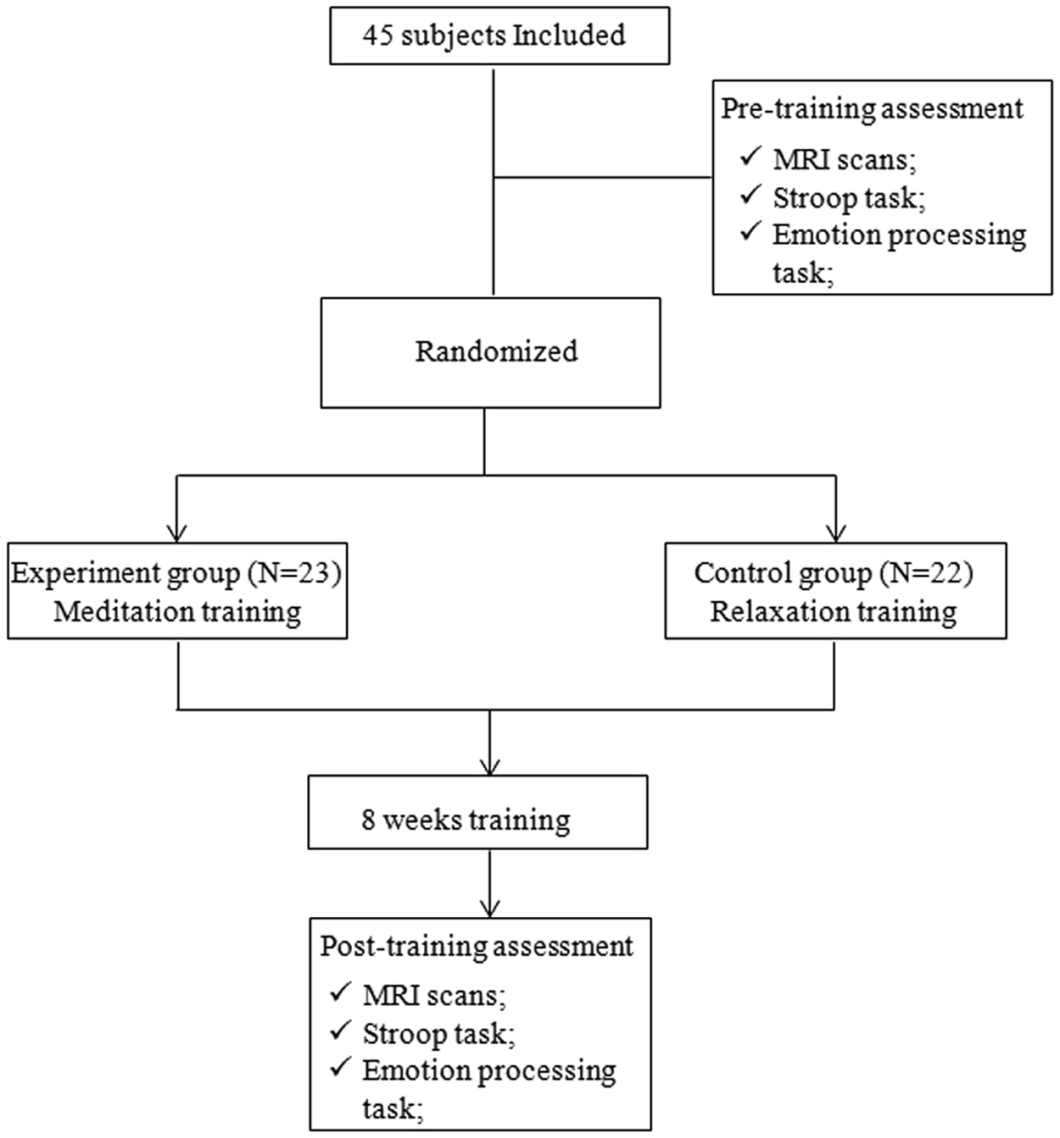
Flowchart of participant flow. Data from 23 subjects from the mediation training group (MT) and 22 subjects from the relaxation training group (RT) were initially entered into the analysis. Amongst these subjects, 4 subjects were excluded due to head motions larger than 3 mm or 3^º^. Thus, 22 subjects for MT and 19 subjects for RT were entered into the final network construction and analysis.

There were no major differences in gender, age or years of education between the meditation training (MT) group and relaxation training (RT) group. The demographic characteristics of the subjects in each group are presented in Table 1. Details regarding the number of participants randomized to each group, and the number of participants entered in the final analysis can be seen in Fig. 1.

**Table 1:** Demographic characteristic of meditation practitioners and control subjects.

	MT	RT	*X* ^2^/*t*, *p*
Gender	16 F/7 M	14 F/8 M	*X* ^2^ = 0.17, *p* = 0.67
Age (years)	64.78 ± 2.71	64.68 ± 2.19	t = 0.13, *p* = 0.89
Education (years)	11.90 ± 3.02	13.89 ± 3.93	t = −1.41, *p* = 0.16
Duration of practice (mins)	710 + 12.89	711.23 ± 15.89	t = 0.13, *p* = 0.70

Note: MT/RT, meditation training/relaxation training group; F/M, female/male.

### Outcome Estimation

#### Neuroimaging assessments

*Global Level* : *Whole brain topology*. No group differences at baseline were found on the primary outcome measures. ANOVA analysis showed that there were no main effects on the group and time factors, while interaction effects were detected in several network metrics, including network cost, global efficiency (*E*glob), local efficiency (*E*loc), shortest path length (*L*p), cluster coefficient (*C*p), network strength (*S*p), and overall modularity (*Q*) (*p* < 0.05) (Table 2). Tests of simple effects suggested that after short term training, the RT group showed increased global efficiency, local efficiency, cluster coefficient, and decreased shortest path length (*p* < 0.05). There were, however, no significant changes before and after short term training in the MT group.

**Table 2:** Global network topology between Time (Pre/Post) and Group (Mediation training, MT, and relaxation training, RT).

Metrics	Main effects	Group F (1, 36), p, η	Interaction effects	Post test	
				MT	RT
	Timing F (1, 36), p, η		Timing * Group F (1, 36), p, η	Post vs Pre	Post vs Pre
Cost	0.02 (0.88), 0.001	1.45 (0.24), 0.04	5.23 (0.02), 0.12	↓	↑
Cost-efficiency	0.01 (0.91), 0.001	1.46 (0.24), 0.03	4.76 (0.03), 0.11	↑	↓
*E*glob	0.05 (0.83), 0.001	1.14 (0.74), 0.04	5.93 (0.02), 0.14	↓	↑
*E*loc	0.01 (0.97), 0.001	1.44 (0. 23), 0.04	4.98 (0.03). 0.12	↓	↑
*L*p	1.30 (0.26), 0.03	0.01 (0.91), 0.001	5.09 (0.03), 0.12	↑	↓
*C*p	1.43 (0.24) 0.03	0.01 (0.92), 0.001	4.19 (0.04), 0.10	↓	↑
*S*p	1.41 (0.24), 0.04	0.04 (0.84), 0.001	5.71 (0.02), 0.13	↓	↑
*Q*	0.11 (0.74), 0.003	0.32 (0.58), 0.01	4.32 (0.04), 0.11	↑	↓

Note: η, effect size; n.s., no significant difference; ↑(↓) compared with pre phrase, network metrics showed increased (decreased) trends after training (Post).

**Figure 2:**
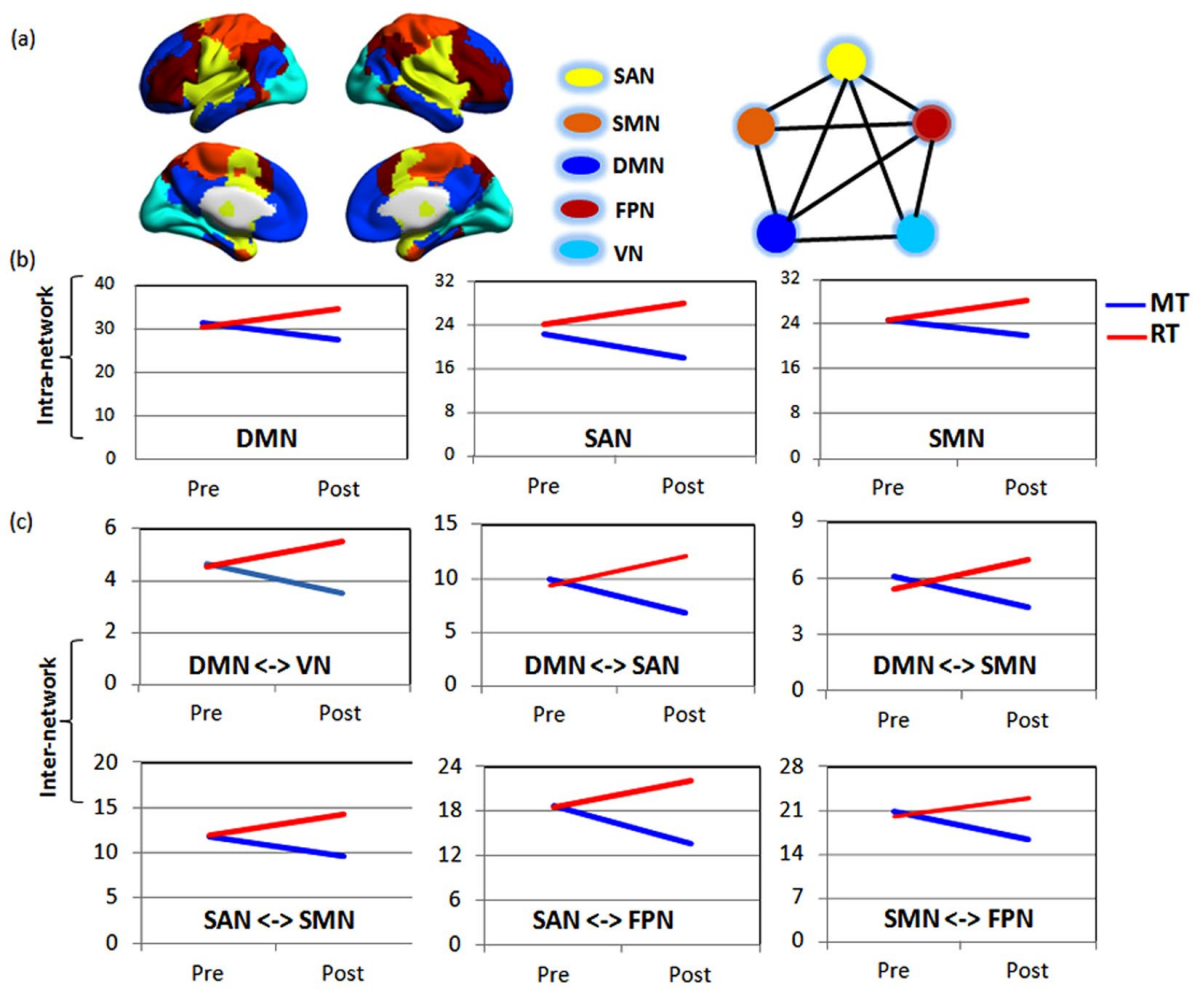
The Effects of training on intermediate modular structure. Five modules were identified for the group-level mean network of pre-test phrase (**a**) including the default mode network (DMN), salience network (SAN), somotomotor network (SMN), fronto-pariteal network (FPN), and visual network (VN). Further statistical analysis revealed significant interactions between timing and group (*p* < 0.05, corrected) on the intra-module and inter-module functional connectivity. Figures (**b** and **c**) show the post test for the intra-module/inter-module functional connectivity showing interaction. The results represented on the brain surface were mapped using the BrainNet viewer^52^.

*Intermediate level* : *Intra* - *module and inter* - *module connectivity profile*. Based on the group-averaged functional connectivity matrixes from the pre-test data, we identified five modules (*Q* = 0.54, *p* < 0.001): default mode network (DMN), salience network (SN), fronto-parietal network (FPN), somatomotor network (SMN), visual network (VN) (Fig. 2a). On the basis of this modular architecture, we applied ANOVA on the connectivity strength within each subnetwork or inter-connectivity strength. No main effects on time and group factors were detected, but interaction effects were found on the network connectivity strength in DMN, SAN, and SMN (*p* < 0.05) (Fig. 2b: see Fig. S2 in Supplementary for detailed results). Further tests of simple effects demonstrated that after training the MT group showed less intra-connectivity in these networks (*p* < 0.05), and the RT group showed increased connectivity strength compared with the baseline phrase (Pre) (Fig. 2b).

No main effects were detected in the inter-network connectivity strength, but group by timing interaction effects were found. Tests of simple effects showed that the DMN exhibited lower connectivity strength with several other modules in the MT group after training, while in the RT group the DMN showed increased connectivity strength to other networks (*p* < 0.05, Fig. 2c).

**Figure 3:**
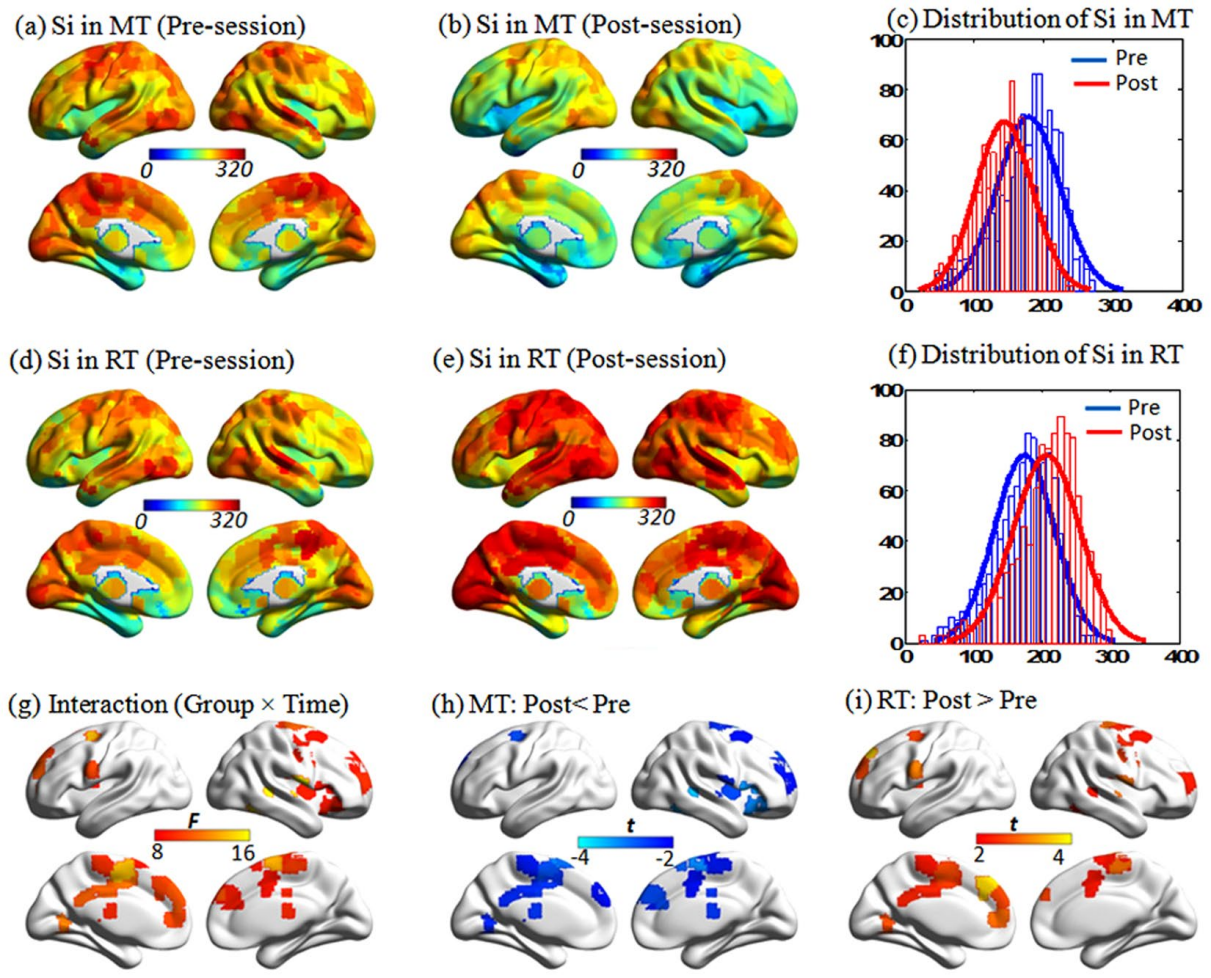
The effects of training on local nodal strength. (**a**,**b**,**d** and **e**) Local nodal strength distribution before and after training. (**c** and **f**) Local nodal strength distribution before and after training. (**g**) ANOVA analysis demonstrated the regions showing interaction effects between Timing and Group (*p* < 0.005). (**h** and **i**) Showed the simple effect test for the mediation training (MT) and relaxation training (RT) group (*p* < 0.05). Of note that nodes showed interaction effects were decreased after training in MT compared with before training, while RT group showed increased nodal strength after training. The results represented on the brain surface were mapped using the BrainNet viewer^52^.

*Local Level* : *Nodal strength*. There were no significant differences between the MT and RT groups at the baseline phase. To localize the regional nodes with training effects, we contrasted the nodal strength for each node between the two groups. The mean nodal strength was heterogeneously distributed over the brain with the most highly connected regions in the prefrontal cortex and posterior parietal and occipital cortices; a common pattern to both groups (Fig. 3a,b,d,e). Nevertheless, 28 ROIs exhibited significant interaction effects between group and time factors (*p* < 0.005, uncorrected). These ROIs predominately encompassed the posterior cingulate gyrus, lateral prefrontal cortex, lateral temporal and parietal cortices, medial temporal lobe, supplementary motor area, bilaterally (Fig. 3g). Tests of simple effects suggested that after short term training, the MT group demonstrated lower nodal connectivity in the left posterior cingulate gyrus, bilateral paracentral lobule, and middle cingulate gyrus (Fig. 3h). While the RT group showed higher nodal connectivity in the right middle temporal gyrus, the left lingual gyrus, bilateral supplementary motor area, and bilateral paracentral lobule (Fig. 3i). Interestingly, these regions were mainly located in the SAN, DMN and SMN.

*Validation and reproducibility*. In general, both the global and nodal results reported in the main text were largely reproducible when: (1) data was pre-processed with 6 mm smoothing kernel; (2) networks were constructed with binary links. The detailed results on the global network metrics and 3D profile for nodal strength are characterized in Table S1 and Figures S3 and S4 in Supplementary.

#### Behavioural Assessments

Detailed results from the Emotion processing task (EPT) have been reported in reports previously produced by our lab (Shao *et al*.^13^). In brief, for the EPT task the MT group showed weaker valence and arousal responses after the 8-week training period (*p* < 0.05), but these measures remained unchanged in the RT group. For the Stroop task, no significant main or interaction effects were detected for either group (*p* > 0.05).

## Discussion

The direct effects of an eight-week meditation training on neural network configuration was studied by employing a longitudinal design and an active control group. Our findings clearly indicate that only the intermediate and local levels of brain connectivity showed changes resulting from the meditation training. At the intermediate level, decreases in intra-connectivity in the default mode network (DMN), salience network (SAN) and somatomotor network (SMN) modules have been shown among the meditation group, as compared to the controls after training. The DMN was also shown to have decreased connectivity strength with other modules in the meditation group. At a local level, the meditation group showed lower nodal strength in the left posterior cingulate gryus, bilateral paracentral lobule, and middle cingulate gyrus post training. These findings highlight the importance of considering meditation-induced neural changes across different organizational levels, and the different paces at which they may occur. On a clinical level, the direction of these network changes is consistent with a movement towards a more self-detached viewpoint; and a more neutralized affective style as we have previously established^13^. The established changes are also indicative of potentially more efficient processing. Together these findings suggest that only 8 weeks of meditation training may have a significant positive impact on the elderly brain and thus further support the view that meditation may be beneficial for the elderly.

The findings reflect no significant changes in graph metrics at a global level in the meditation group, which is consistent with Jao *et al*.’s study^17^ that similarly found no differences in global network organization when comparing meditative and resting states in expert meditators. It seems that, contrary to our expectations, eight weeks of meditation may not have improved the small worldness of the brain organization of the elderly. There are several potential explanations for these findings. Firstly, it is possible that the lack of changes reflect a conservative effect of meditation on the brain. That is, rather than the network organizational structure showing reductions in small-worldness as may be expected in elderly populations^15^, the network topology remained the same which may suggest that the meditation training preserved the global network topology. Future studies may wish to employ a further non-active (no training) control group to examine how the network organizational structure of elderly individuals changes over a period as short as 8-weeks to assess the validity of this proposal. Another possible explanation is that the duration of training may have been too brief to reveal observable changes at a global level, which would suggest that network configuration changes may occur at different paces^18^. However, given the findings of Jao *et al*.^17^ it seems most likely that meditation training simply does not impact network organization at a global level and instead has more localized effects. A study employing further post-training follow ups would be needed to confirm this proposal, and establish how exactly network organizational structure may change further with increasing expertise.

Our findings regarding reductions in intra-network connectivity within the DMN after meditation are consistent with a recent study which found that functional connectivity between two components of the DMN, as identified by Independent Component Analysis, was negatively correlated with mindfulness in novice meditators, who completed a two-week attention training in breathing (Doll *et al*.^23^). Our findings are also in line with several studies which have found reduced connectivity within certain regions of the DMN in meditation experts compared to novices^19–21^. Given the DMN’s established role in self-referential thinking^22^, these findings are thought to reflect a movement towards an increasingly self-detached viewpoint associated with meditation^13, 23^. The DMN also plays a role in determining the affective relevance of a specific stimulus. This reduced connectivity between regions of the DMN therefore may also reflect changes in how relevant to self an individual considers a stimulus^23, 24^. This explanation is supported by our previously reported behavioural data which suggests meditation training ‘neutralizes’ the affective processing of both positive and negative stimuli in the elderly^13^. Several other studies, however, have found increased connectivity within specific regions of the DMN^25–28^. As there have been considerable differences in methodologies across the aforementioned studies, they all suggest intra-connectivity with the DMN may vary largely depending on factors including the specific regions examined, analytical approach (e.g. region vs. whole brain), meditation type and state (resting or meditative), rather than offering contradictory findings respectively. This highlights the importance of methodological specificity in examining intra network connectivity in relation to meditation.

The salience and somatomotor networks have received considerably less attention relative to the DMN in relation to meditation research. Farb, *et al*.^29^ compared neural activity and connectivity across three tasks (one focused on interoceptive attention and the other two focusing on other forms of attention) among individuals who had completed eight-week meditation training and waitlisted controls. In the meditation group, task-independent increased connectivity was found between the right posterior insula and the right anterior insula (regions in the SAN) after MBSR training. This finding suggested increased interoceptive awareness, regardless of external stressors, which is a key goal of meditation^29^. As this study focused on task-related activity whilst our study focuses on resting-state activity, it could be argued that these differences reflect differences across states, as has been demonstrated by Jao, *et al*.^17^.

Consistent with these findings of lower intra-connectivity within key meditation networks, our study also revealed lower nodal strength in regions, including the left posterior cingulate gyrus, bilateral paracentral lobule, and middle cingulate gyrus post-training in the meditation group. These findings indicate lower interconnectivity of these regions with other nodes^30^. These regions are found primarily within the DMN, SAN and SMN. The posterior cingulate gyrus, for example, is a key hub of the DMN^31^. This suggests that targeting key hubs within these networks can be one manner in which meditation may impact the brain^30^.

Our findings of lower connectivity between the DMN and several other networks (including the SAN, SMN and VN) in the meditation group are in line with previous literature. A number of studies have examined connectivity between the DMN and SAN at resting state, for example, and found indications of reduced connectivity with these modules^20, 32^. The aforementioned study by Doll, *et al*.^23^, for instance, found an anti-correlation between abilities of mindfulness and inter-intrinsic functional connectivity (IFC) among SAN, DMN and the central executive network (CEN) after two weeks of daily twenty-minute meditation. Given the DMN’s established role in mind wandering^33^, this lesser connectivity with the SAN may indicate increased awareness to mind wandering^23^. In general, this lesser connectivity between key modules involved in meditation may be interpreted as a stronger distinction between networks that may result in better effective connectivity^23, 34^, and thus potentially more efficient processing. A possible focus for future studies would be to examine relationships between such measurements (e.g. processing speed) and inter-network connectivity within the DMN.

Our findings must be considered in light of several limitations. Firstly, although we have explained some of our results in terms of the differences associated with different levels of expertise in meditation, our study has only employed one post-training follow-up. Future studies employing further post-training follow-up points would be required to confirm this explanation. Secondly, we have focused solely on one form of meditation. There is, however, evidence wherein distinct neural activities may be attributed to the different types of meditation^35^. Thus, it would be interesting to explore the ways various meditation types influence brain network configuration. Thirdly, whilst our training groups were matched on several measures, future studies may wish to employ a more comprehensive range of cognitive and affective instruments to ensure greater matching across groups. Fourth, although our study aimed at examining topological changes in the elderly specifically, future studies could help further clarify the impact of meditation on the brain’s architecture across the lifespan by employing a wider age range of participants. Finally, given the exploratory nature of the study, for the intermediate and global level analyses we used a fairly lenient correction method (1/N, N is the test counts) to control for multiple comparisons^36, 37^ and no correction method was employed for the local level analyses. The results should thus be interpreted with caution and future studies may wish to employ larger sample sizes to allow for more stringent correction methods.

Overall, this study examined the direct effects of short-term training in meditation on functional network organization amongst the elderly. It was found that an eight-week meditation training caused significant changes to the neural network configuration, with lower intra-connectivity found in key meditation networks including the DMN, SAN and SMN network. These findings are speculated to reflect a movement towards a more self-detached viewpoint as a result of the meditation practice. Less inter modular connectivity, particularly with the DMN, was also found, which suggests a greater distinction amongst networks and thus, according to previous literature, potentially better processing. Finally, no global level changes were found in the meditation group suggesting meditation may have a more localized influence on the brain, or that network changes may occur at different paces thus highlighting the need for further longitudinal studies to assess network changes at various time points. Together these findings highlight the importance of considering the neural impact of meditation at various topological levels and the varying pace at which these changes may occur, and provide further support for short-term meditation as a potentially beneficial method of mental training for the elderly that warrants further investigation.

## Method

### Study Design

The Institutional Review Board of The University of Hong Kong (HKU) and the Hospital Authority approved this study; and the methods were carried out in accordance with the approved guidelines. All participants gave their informed consent for participation. Participants were randomly assigned to receiving either meditation training (MT, n = 23; 16 females; mean age = 64.78 ± 2.71 years, range = 60–68 years) or relaxation training (RT, n = 22; 14 females; mean age = 64.68 ± 2.19 years, range = 61–69 years). All participants attended two data collection sessions (pre and post). In the pre-training session, participants were first screened for the exclusion criteria. Participants then completed the TONI-III IQ test and the HADS. The resting-state fMRI and structural MRI protocols were then conducted for the participants and behavioural measures were taken. Participants then received 8 weeks of either meditation or relaxation training, as detailed below. Within 3 weeks following the completion of training, participants underwent post-training imaging and behavioral assessments, which were performed under the same protocols as for the pre-training assessment. After study completion, participants were debriefed, thanked and reimbursed 800 Hong Kong dollars.

### Study Population

Forty-five healthy elderly adults with normal general intelligence and no prior meditation or relaxation training experience were recruited through community newsletters. Demographically, the two groups were matched for age (*p* = 0.89), sex (*p* = 0.68), and years of education (*p* = 0.17). Furthermore, the meditation and relaxation groups performed similarly on the Test of Nonverbal Intelligence, third edition (TONI-III) (*p* = 0.91), and showed comparable levels of anxiety (*p* = 0.48) and depression (p = 0.66), as measured by the Hospital Anxiety and Depression Scale (HADS). All participants were right-handed, as assessed with the Edinburgh Handedness Inventory; had normal or corrected-to-normal vision and hearing; reported no history of major physical illnesses, neurological or psychological conditions, such as substance abuse, psychotic disorders, or affective disorders; and were suitable to enter a magnetic resonance image (MRI) scanner. In order to manage expectations across participant groups, both groups were led to believe that both interventions would lead to significant improvements. Specifically, at the beginning of the study, participants were informed that the purpose of the study was to investigate whether meditation and relaxation practice may promote healthy aging through slowing down brain degeneration and boosting immune functions, and that both interventions would lead to improvements. In the event that participants further questioned the benefits/improvements of both interventions the purpose of the study was restated and participants were again told that both interventions have several benefits (e.g. more relaxation).

### Study Interventions

Participants received either attention-based compassion meditation training or relaxation training in a group setting for 8 weeks. Relaxation training is a reliable active control for studying the effects of meditation as both forms of training involve similar experiences^13^. Furthermore, both training programs were designed to follow largely the same structure to minimize differences. Each type of training involved 22 classes: 11 taught sessions, 10 group practice sessions and one intensive 3-hour session. Each of the taught and practice sessions lasted 1.5 hrs. Each participant therefore completed a total of 34.5 hrs training. Each taught class started with a guided meditation or relaxation practice for ~30 mins, didactic teaching for ~45 mins and ended with another guided practice for ~15 min. The structure of the group practice sessions was largely similar although meditation/relaxation sessions were guided by videotapes, and participants did didactic revision. All group practice sessions were videotaped. The 3 hrs intensive session was structured as follows: brief introduction (15 mins), guided meditation/relaxation (1 hr), tea break/noble silence (15 mins), meditation/relaxation practice (45 mins), Q & A (30 mins) and meditation/relaxation (15 mins). Participants were asked to practice outside of class on a daily basis for a minimum of 20 minutes (on days without sessions), and their practice durations were recorded. The total self-practice time was comparable between the meditation (average 710 mins) and relaxation groups (average 711 mins). Further details of the training programs are described below.

#### Meditation training

The meditation training was conducted by an experienced meditator with 14 years of meditation practice and 4 years of teaching experience. Meditation participants were taught to (1) cultivate mindfulness through paying attention to the surrounding sounds and one’s own breathing, feelings and sensations on the present moment, (2) try to apply non-judgmental and ‘acceptance’ attitudes on thoughts, feelings and sensations, (3) try to detach from a self-referential framework and observe one’s own thoughts and feelings from an outsider’s perspective, and (4) try to cultivate compassion and kindness towards self, family members, friends, strangers, and other living beings.

#### Relaxation training

Relaxation training was conducted by a registered clinical psychologist with 4 years of teaching experience. Relaxation participants were taught diaphragmatic breathing, progressive muscle relaxation and imagery relaxation techniques aimed at enhancing body awareness and reducing body tension.

### Study Outcomes

#### Neuroimaging assessments

*Data acquisition*. All MRI datasets were obtained on a 3T Philips MR scanner with the use of a 12-channel phased-array receiver-only head coil. The R-fMRI datasets were acquired using a gradient echo EPI sequence with the following parameters: repetition time (TR) = 2000 ms, echo time (TE) = 30 ms, flip angle = 90^o^, field of view (FOV) = 224 × 224 mm^2^, data matrix = 64 × 64, thickness = 4 mm, 32 transverse slices covering the whole brain, and 180 volumes acquired in 6 mins. During the R-fMRI scan, each subject was asked to keep their eyes closed but not to fall asleep and to relax their minds but not to think about anything in particular. We also collected high-resolution anatomical images of individuals using a T1-weighted three-dimensional volumetric magnetization-prepared rapidly acquired gradient-echo sequence: 192 slices; TR = 6.88 ms; TE = 3 ms; FA = 8^o^; slice thickness = 1 mm; no gap; matrix = 240 × 240; and FOV = 240 × 240 mm^2^.

*Data preprocessing*. The R-fMRI data were preprocessed using SPM8 (http://www.fil.ion.ucl.ac.uk/spm/) and DPARSF^38^. For each subject, the first five volumes of the R-fMRI dataset were discarded to allow for MR signal equilibrium, leaving 175 volumes for further analysis. The remaining images were then corrected through the use of slice timing to control for the acquisition time delay between slices within the same TR, realigned to the first volume to correct the inter-TR head motions, and spatially normalized to a standard MNI template and re-sampled to a voxel size of 3 × 3 × 3 mm^3^. In accordance with previous studies, no spatial smoothing was applied^16, 39^. Finally, we performed band-pass filtering for each voxel in the frequency of 0.01~0.01 Hz to reduce low-frequency, drift and high-frequency physiological noise. The R-fMRI data for each subject were checked for head motion. Four subjects were excluded according to the criteria that the translation and rotation of head motion in any direction were not more than 3 mm or 3°. If Power frame displacement (FD) was found to be greater than 1, that time point was deemed a ‘bad’ time point, and the time points before and after that bad time point were scrubbed using each of the bad time points as a repressor^40^. Finally, a total of 41 subjects were entered into the data analysis, 22 subjects in meditation group, and 19 subjects in relaxation group (See Fig. 1).

*Network construction*. Using nodes and edges, as the basic elements of a network, we deployed the GRETNA toolbox^41^ to construct the brain functional network for each subject according to the AAl-1024 atlas, which consists of 1024 regions. We calculated the time series for each ROI by averaging the time courses of all the voxels within a ROI, and performed a linear regression to remove the effects of the following covariates from each voxel’s time course: signals from the brain white matter and cerebrospinal fluid as well as 24-parameter head-motion profile. With respect to global signals, we referred to the Global Negative Index (GNI)^42^ as an indicator as to whether global signal regression was needed for the current study. This index recommends not performing global signal regression analysis when participants GNI are 3 or above. After examination of GNI profiles, which were estimated using the publicly available Matlab code (By Chen Gang, https://www.mathworks.com/matlabcentral/fileexchange/36864-determine-the-necessity-for-global-signal-regression) we found the majority of participants to have a GNI greater than 3. Furthermore a paired t-test revealed that there were no significant difference pre and post intervention (*p* < 0.42). Based on these observations, we thus decided not to perform global signal regression analysis on this dataset. The GNI profile of each subject can be seen in Fig. S1 in Supplementary.

For each subject, we first used the residuals of the time series for each ROI to calculate a Pearson’s correlation coefficient and the significance level (i.e., *p* value) of a given inter-regional correlation. Then we obtained a 1024 × 1024 symmetric correlation matrix and the corresponding p value matrix for each subject. To de-noise spurious correlations, we retained only those correlations whose corresponding p values passed through a statistical threshold of *p* < 0.05 (Bonferroni correction); otherwise, we considered there to be no functional connectivity between the two regions. Notably, negative correlations were also excluded in this study because of the ambiguities in their interpretation^43^ and detrimental effects on test–retest reliability^44^. Finally, we obtained a weighted 1024 × 1024 FC matrix, which was used to conduct the subsequent analysis for each subject.

*Network metrics*. All network measures used in this study, including global efficiency, local efficiency, nodal efficiency, are explained in the context of a weighted network *G* with *N* nodes and *K* edges.

#### Global network parameters

Cost. For a network (graph) *G* with *N* nodes, the wiring cost is defined by the ratio between actual links of the network and the possible links of this network.

Network strength. For a network (graph) *G* with *N* nodes and *K* edges, we calculated the strength of *G* as:

1 $${S}_{{\rm{p}}}(G)=\frac{1}{N}\sum _{i=1}^{N}{S}_{i}$$

where *S*_i_ is the sum of the edge weights w_ij_ (i.e., correlation coefficients) linking to node *i*. The strength of a network is the average of the strengths across all the nodes in the network.

Global efficiency. The global efficiency of G can be computed as:

2 $${E}_{{\rm{glob}}}(G)=\frac{1}{N(N-1)}\sum _{i=1}^{N}\sum _{j\ne 1}^{N}\frac{1}{{L}_{ij}}$$

where *L*_ij_ is the shortest path length between node *i* and *j* in G. The path length between node *i* and node *j* is defined as the sum of the edge lengths along this path, where each edge’s length was obtained by computing the reciprocal of the edge weight, 1/W_ij_. The shortest path length *L*_ij_ between node *i* and *j* is the length of the path with the shortest length between the 2 nodes.

Local efficiency. The local efficiency of G is measured as^45^:

3 $${E}_{{\rm{loc}}}(G)=\frac{1}{N}\sum _{i\in G}{E}_{{\rm{glob}}}({G}_{i})$$

where *E*_loc_(G) is the global efficiency of G_(*i*)_; the subgraph composed of the neighbors of the node *i* (i.e., nodes linked directly to node *i*). The local efficiency measures the fault tolerance of the network, which indicates the capability of information exchange for each subgraph when the index node is eliminated.

Shortest path length. The weighted shortest path length *L*_p_ is defined as

4 $${L}_{{\rm{p}}}(G)=\frac{1}{N(N-1)}\sum _{i\ne j\in G}{L}_{{\rm{ij}}}$$

It is measured by using a “harmonic mean” geodesic distance between all pairs. It reflects the optimal path of information transfer from node *i* to *j* node and then economizes the cost of information transfer through the shortest path. *L*_p_ quantifies the ability of parallel information propagation or global efficiency of a network.

Cluster coefficient. The cluster coefficient *C*_p_ is defined as

5 $${C}_{{\rm{p}}}(G)=\frac{1}{N}\sum _{i\in G}\frac{{K}_{i}}{{D}_{i}({D}_{i}-1)/2}$$

K_i_ is the number of edges in G(*i*); the subgraph consisting of the neighbors of node *i*.

*D*_i_ is the degree, or the number of edges connected to the node *i*. *C*_p_ measures the cliquishness of a network.

Cost-efficiency. In our current study, we followed previous work^14^ and defined the cost-efficiency as the ratio between global efficiency (*E*glob) and cost.

#### Intermediate level parameters

Modular analysis. Modules are defined as sets of nodes that are densely linked with each other and less so with other nodes in the network (that is, other modules). The modularity, *Q*, is defined as

6 $$Q(G)=\sum _{s=1}^{{N}_{M}}\,[\frac{{w}_{s}}{W}-(\frac{{W}_{s}}{2W})]$$

while *N*_*M*_ is the number of modules, *W* is the total weight of the network, *W*_*S*_ is the sum of the connectional weights nodal strength in module s (see the definition of nodal strength, *S*_*i*_). *Q* quantifies the difference between the weight of intra-modular edges in the real network and that of random networks^46^. To maximize *Q* value resulting in the best possible modular partitions, we used the spectral optimization algorithm proposed by Newman^47^ and reported the maximized value of *Q* for the brain networks. Higher values of *Q* indicate greater functional specialization of a brain network.

In this study, we estimated the most representative group-level modular partitions of all subjects at first scanning. First, we averaged each edge weight across individuals to obtain the group-averaged weighted FC matrix for each group. Second, based on this group-mean FC matrix, we used a nonparametric sparsification method^48^ to extract the backbone network using *p* < 0.05. In this calculation, we selected those locally significant edges which could not be explained by random variations to form the backbone networks. Finally, the backbone network was used to identify the modular partition that captured underlying connectivity patterns for all subjects in the present study.

#### Local level parameters

Nodal strength. (*S*_*i*_) is a simple but test–retest reliable measure to characterize nodal centrality^49^ and is calculated as the sum of connectional weights (i.e., correlation coefficients) across all edges that directly link to a given node.

### Behavioural assessments

The Stroop task^50^ was employed as a measure of non-affective attentional control and the Emotional processing task^35^ was used to assess affective style. Further details of these tasks can be found in our previous reports^13, 50^.

## Sample Size and Statistical Analysis

### Between-Group Differences

For the final brain network analysis, data from 41 subjects was analysed (MT = 22, RT = 19). Any statistical group differences at baseline were explored with ANOVAs, while descriptive statistics were used to explore group differences on nominal measures such as gender. Overall recovery between baseline and outcome assessment was analyzed as the factor ‘time’ in a repeated measurements ANCOVA for each of the outcome variables. To examine the training effects on the network metrics with training, we used ANOVA with time phrase (Pre, Post) as within factor, and Group (MT. RT) as between factor. For the global and intermediate analyses, the significance level was corrected with a false-positive correction *p* = (1/N)^36, 37^ where N is test counts. Given the exploratory nature of this study, and the number of tests run (1024) to examine nodal strength, it was decided the use of a more lenient threshold (*p* < 0.005) would be acceptable for local level analyses. All analyses were controlled for age, gender and years of education.

### Robustness analysis

Different preprocessing strategies may affect the calculated network parameters of brain network^51^. Therefore, to test the robustness of the findings, we re-analysed the data using two additional pre-processing strategies, (1) Smoothing, and (2) binary network, as defined below. In the smoothing strategy, we constructed brain networks using the functional signals after smoothing (FWHW = 6 mm) the functional images. Finally, we constructed the binary functional network in which functional connectivity values passed through a statistical threshold of *p* < 0.01 (FDR corrected).

## Electronic supplementary material

Supplementary Information
