# Supplementary material for: A longitudinal study of the effect of short-term meditation training on functional network organization of the aging brain
Source: Sci Rep. 2017 Apr 4;7:598. doi: 10.1038/s41598-017-00678-8 (PMC5428857; doi:10.1038/s41598-017-00678-8)
Supplement: Supplementary file 1 — Supplementary Information [file 41598_2017_678_MOESM1_ESM.pdf]

Supplementary

A longitudinal study of the effect of short-term meditation training on functional network organization of the aging brain

Francesca A. Cotier [1,2†], Ruibin Zhang [1,2†], Tatia, M.C. Lee [1,2,3,4*]

[1] Laboratory of Neuropsychology, The University of Hong Kong, Hong Kong.
[2] Laboratory of Cognitive Affective Neuroscience, The University of Hong Kong, Hong Kong.
[3] The State Key Laboratory of Brain and Cognitive Sciences, The University of Hong Kong, Hong Kong.

[†] Both authors contributed equally to this work

***Correspondence to:**
Tatia M.C. Lee, May Professor in Neuropsychology
Address: Room 656, Laboratory of Neuropsychology, The Jockey Club Tower, The University of Hong Kong, Pokfulam Road, Hong Kong
Tel.: (852) 3917-8394
E-mail address: tmclee@hku.hk

Table and Figure legend

Table S1 Global network topology between Timing (Pre/Post) and Group (Mediation training, MT, and relaxation training, RT) under different data preprocessing strategies.

Fig S1 Global negative index.

Fig. S2 Interaction effects of sub-network connectivity profile (Group * Time). Default mode network (DMN), salience network (SAN), somotomotor network (SMN), fronto-pariteal network (FPN), and visual network (VN).

Fig. S3 Validation analysis with data pre-processing with 6mm smoothing kernel. (a,b, d, and e) Local nodal strength distribution before and after training. (c and f) Local nodal strength distribution before and after training. (g) ANOVA analysis demonstrated the regions showing interaction effects between Timing and Group ($p<0.005$). (h and i) showed the simple effect test for the mediation training (MT) and relaxation training (RT) group ($p<0.05$). Of note that nodal showed interaction effects were decreased after training in MT compared with before training, while RT group showed increased nodal strength after training. The results represented on the brain surface were mapped using the BrainNet viewer [(Xia et al., 2013)].

Fig. S4 Validation analysis with network construction using binary links. (a,b, d, and e) Local nodal strength distribution before and after training. (c and f) Local nodal strength distribution before and after training. (g) ANOVA analysis demonstrated the regions showing interaction effects between Timing and Group ($p<0.005$). (h and i) showed the simple effect test for the mediation training (MT) and relaxation training (RT) group ($p<0.05$). Of note that nodal showed interaction effects were decreased after training in MT compared with before training, while RT group showed increased nodal strength after training. The results represented on the brain surface were mapped using the BrainNet viewer [(Xia et al., 2013)].

Table S1 Global network topology between Timing (Pre/Post) and Group (Mediation training, MT, and relaxation training, RT) under different data preprocessing strategies.

Metrics	Main effects		Interaction effects	Post test	
				MT	RT
	Timing	Group	Timing * Group	Post vs Pre	Post vs Pre
Smoothing kernel with 6mm					
Cost	0.18 (0.674)	2.05 (0.159)	5.31 (0.026)	↓	↑
Cost-efficiency	0.13 (0.718)	1.53 (0.428)	2.26 (0.14)	↑	↓
Eglob	0.26 (0.615)	2.01 (0.164)	5.21 (0.027)	↓	↑
Eloc	0.89 (0.351)	2.36 (0.132)	3.27 (0.076)	↓	↑
Lp	0.11 (0.738)	1.67 (0.204)	4.19 (0.047)	↑	↓
Cp	0.47 (0.500)	2.26 (0.140)	3.28 (0.08)	↓	↑
Sp	0.34 (0.566)	2.11 (0.154)	5.29 (0.027)	↓	↑
Q	0.26 (0.614)	0.54 (0.466)	5.34 (0.026)	↑	↓
Binary network					
Cost	0.21 (0.647)	1.89 (0.176)	5.36 (0.026)	↓	↑
Cost-efficiency	0.16 (0.689)	1.52 (0.222)	1.89 (0.177)	↑	↓
Eglob	0.11 (0.740)	1.78 (0.190)	5.58 (0.023)	↓	↑
Eloc	0.14 (0.712)	1.82 (0.185)	3.54 (0.067)	↓	↑
Lp	0.04 (0.849)	1.50 (0.228)	4.60 (0.038)	↑	↓
Cp	0.33 (0.567)	1.86 (0.179)	3.01 (0.090)	↓	↑
Sp	0.21 (0.647)	1.89 (0.177)	5.36 (0.025)	↓	↑
Q	0.32 (0.573)	0.48 (0.493)	4.95 (0.032)	↑	↓

Note: n.s., no significant difference; ↑(↓)compared with pre phrase, network metrics showed increased (decreased) after training (Post).

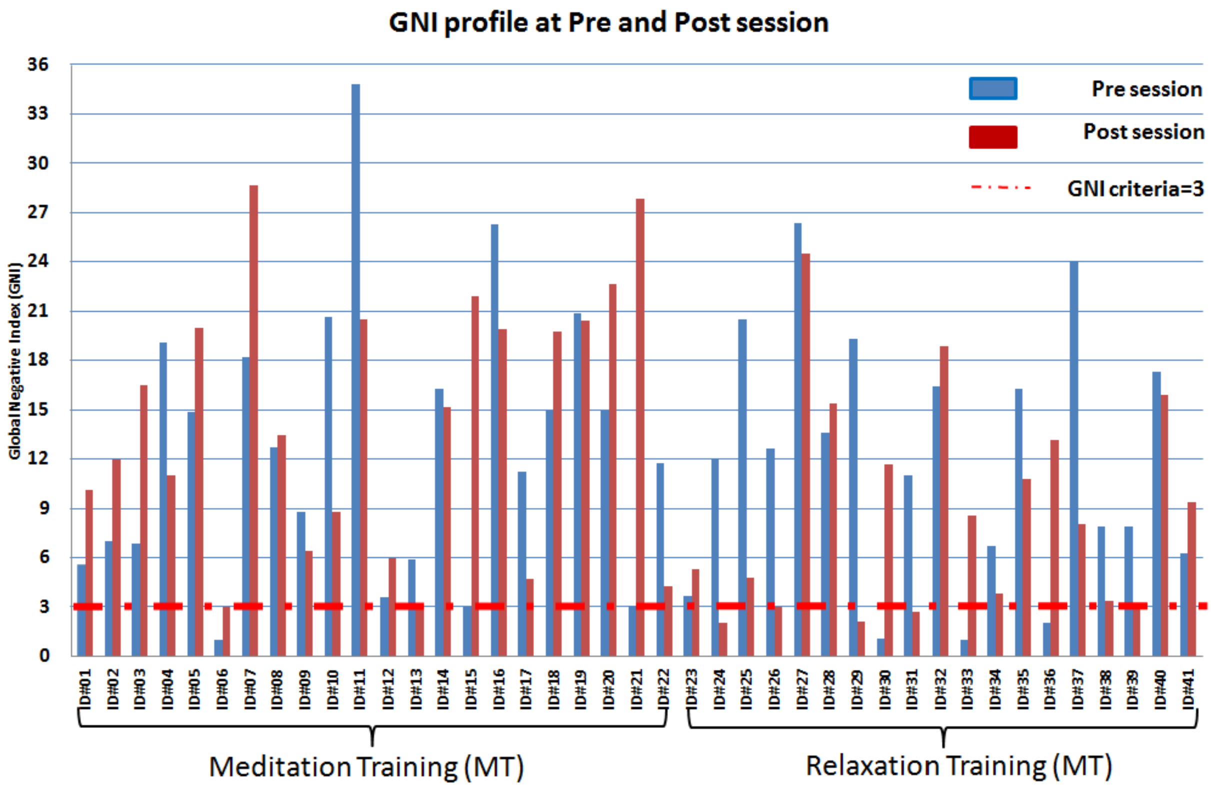

Fig S1 Global negative index (GNI).

	DMN	VN	SAN	SMN	FPN
DMN	**F(1,36)=4.0, p=0.04, η=0.1**				
VN	**F(1,36)=5.67,p=0.02 , η=0.13**	F(1,36)=1.15, p=0.29, η=0.03			
SAN	**F(1,36)=5.8, p=0.02, η=0.13**	**F(1,36)=5.22, p=0.03, η=0.123**	**F(1,36)=7, p=0.01, η=0.16**		
SMN	**F(1,36)=5.33, p=0.03, η=0.13**	**F(1,36)=2.65, p=0.11, η=0.08**	**F(1,36)=7.00, p=0.01, η=0.16**	**F(1,36)=4.00, p=0.04, η=0.10**	
FPN	**F(1,36)=4.39, p=0.04, η=0.11**	**F(1,36)=5.61, p=0.02, η=0.13**	**F(1,36)=4.58, p=0.03, η=0.11**	**F(1,36)=4.39, p=0.04, η=0.11**	F(1,36)=2.5, p=0.13, η=0.06

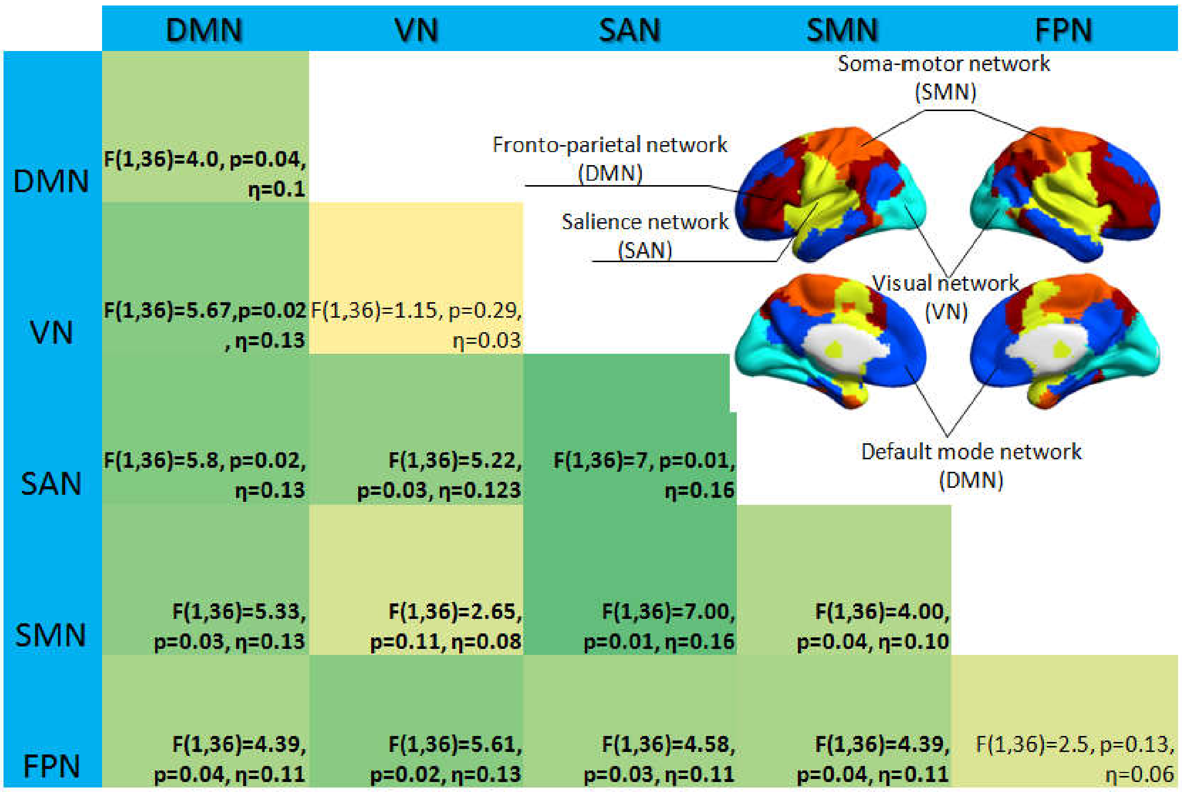

Fig. S2 Interaction effects of sub-network connectivity profile (Group * Time). Default mode network (DMN), salience network (SAN), somotomotor network (SMN), fronto-pariteal network (FPN), and visual network (VN).

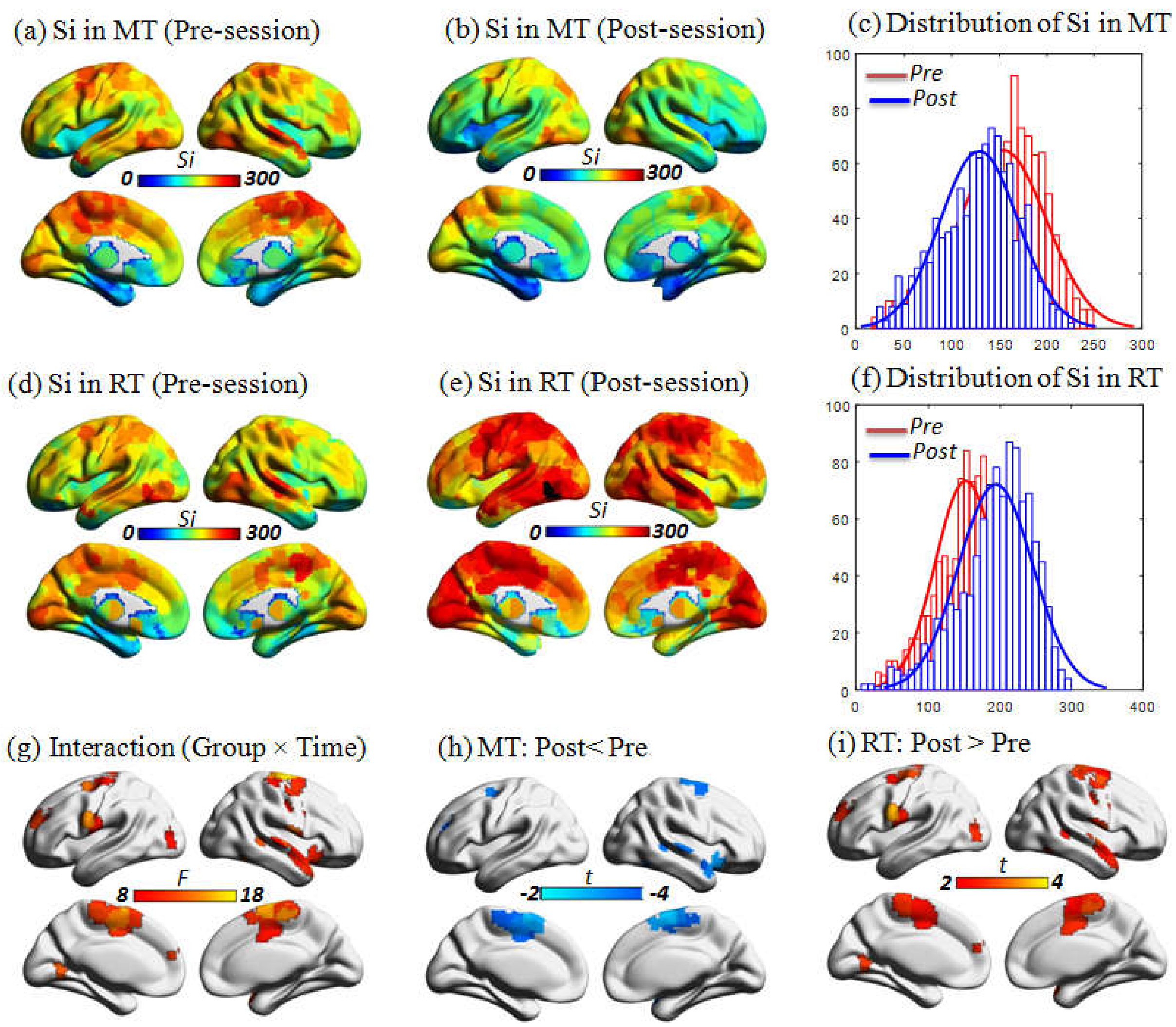

Fig. S3 Validation analysis with data pre-processing with 6mm smoothing kernel. (a,b, d, and e) Local nodal strength distribution before and after training. (c and f) Local nodal strength distribution before and after training. (g) ANOVA analysis demonstrated the regions showing interaction effects between Timing and Group (p<0.005). (h and i) showed the simple effect test for the mediation training (MT) and relaxation training (RT) group (p<0.05). Of note that nodal showed interaction effects were decreased after training in MT compared with before training, while RT group showed increased nodal strength after training. The results represented on the brain surface were mapped using the BrainNet viewer (Xia et al., 2013).

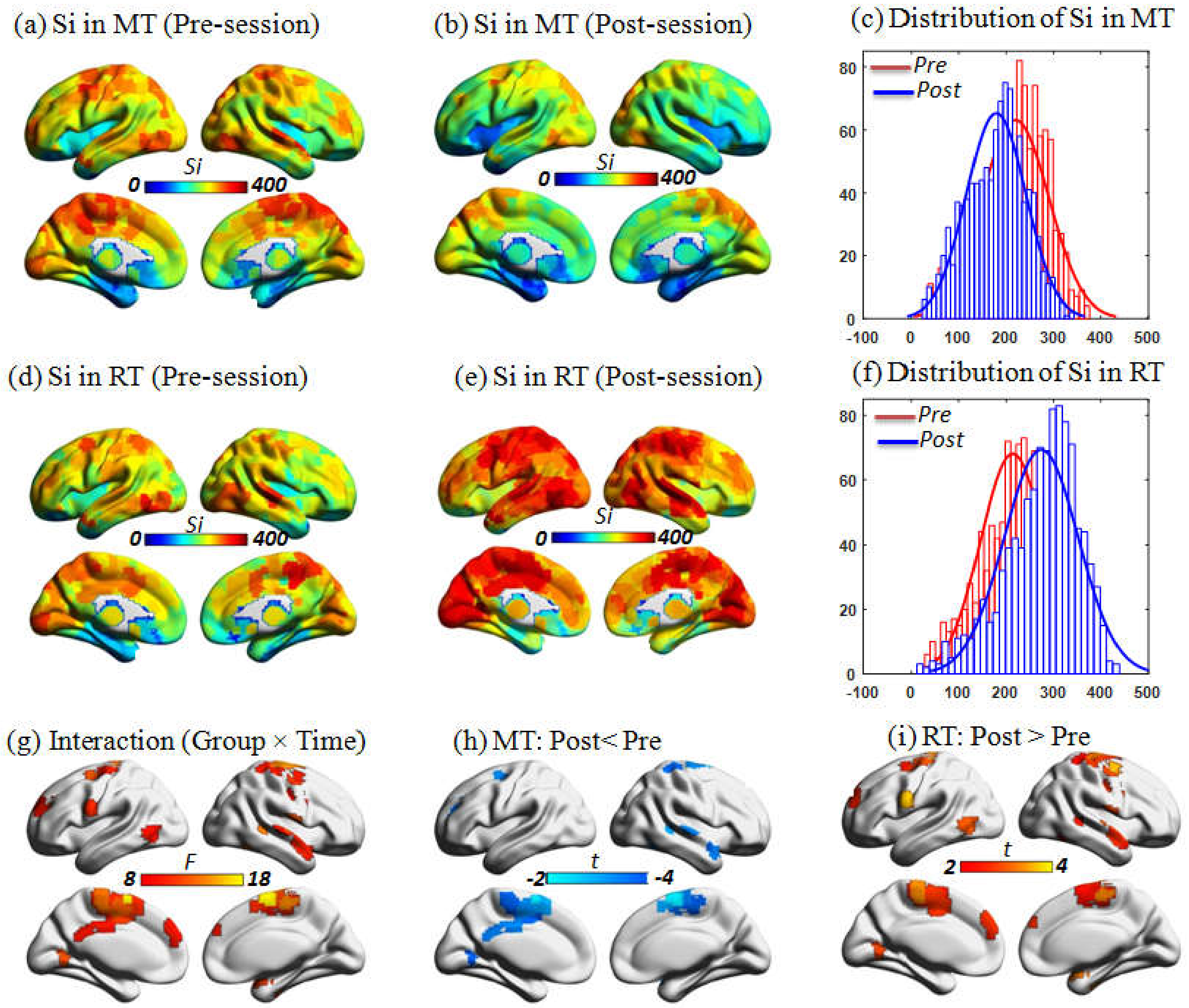

Fig. S4 Validation analysis with network construction using binary links. (a,b, d, and e) Local nodal strength distribution before and after training. (c and f) Local nodal strength distribution before and after training. (g) ANOVA analysis demonstrated the regions showing interaction effects between Timing and Group (p<0.005). (h and i) showed the simple effect test for the mediation training (MT) and relaxation training (RT) group (p<0.05). Of note that nodal showed interaction effects were decreased after training in MT compared with before training, while RT group showed increased nodal strength after training. The results represented on the brain surface were mapped using the BrainNet viewer [(Xia et al., 2013)].
